# Crystal structure of 8-hex­yloxy-2-[(*Z*)-2-(naph­thal­en-2-yl)ethen­yl]quinoline

**DOI:** 10.1107/S2056989022006740

**Published:** 2022-07-05

**Authors:** Xiaozhou Liu, Lu Wang, Ying Feng, Deliang Cui, Zhi Liu

**Affiliations:** a State Key Laboratory of Crystal Materials, Shandong University, Jinan 250100, Shandong Province, People’s Republic of China; University of Durham, England

**Keywords:** π-conjugated, naphthalene derivative, quinoline derivative, π–π inter­actions, intra-mol­ecular hydrogen bond, γ-packing, crystal structure

## Abstract

In the title mol­ecule, C_27_H_27_NO, the naphthalene and quinoline groups are both planar and subtend a dihedral angle of 15.47 (7)°. They are nearly coplanar with the *cis*-vinyl bridge and the hex­yloxy chain, which adopts an all-*trans* conformation, resulting in transannular bifurcated intra­molecular C—H⋯N,O contact. The crystal structure features γ-packing of the aromatic moieties, while the parallel packing of alkyl chains resembles that of alkanes.

## 1. Chemical context

In recent decades, π-conjugated organic mol­ecules with donor–acceptor architectures have received considerable attention regarding their diverse applications in organic optoelectronics and electronics, for example in non-linear optics and as organic semiconductors (Rao *et al.*, 2010 ▸; Siram *et al.*, 2011 ▸; Wang *et al.*, 2015 ▸; Zhang *et al.*, 2015 ▸). As for vinyl-bridged donor–acceptor mol­ecules incorporating naphthalene as a donor and quinoline as an acceptor, the poor solubility (which hinders purification and processibility) is due to the good mol­ecular coplanarity (Ishikawa & Hashimoto, 2011 ▸). The introduction of long substituents into quinoline or naphthalene cores is an effective method of solving this problem. Hex­yloxy-substituted donor–acceptor mol­ecules based on naphthalene and quinoline are a promising class owing to their satisfactory solubility. Moreover, the introduction of alkyl substituents of suitable length can not only increase the capacity for self-assembly, but also improve carrier mobility (Garnier *et al.*, 1993 ▸; Halik *et al.*, 2003 ▸). The title compound (**1**) was synthesized by a Wittig reaction and has been shown by single-crystal X-ray diffraction analysis to be a rare example of a stilbene-like donor–π–acceptor (*D*—π⋯*A*) type mol­ecule with a *cis* configuration, and the first structurally characterized *cis*-naphthalene-C=C-quinoline derivative. The *D*—π⋯*A* structure is known to favour high-intensity two-photon absorption (Lv, Xu, Cui, *et al.*, 2021 ▸; Lv, Xu, Yu, *et al.*, 2021 ▸). 
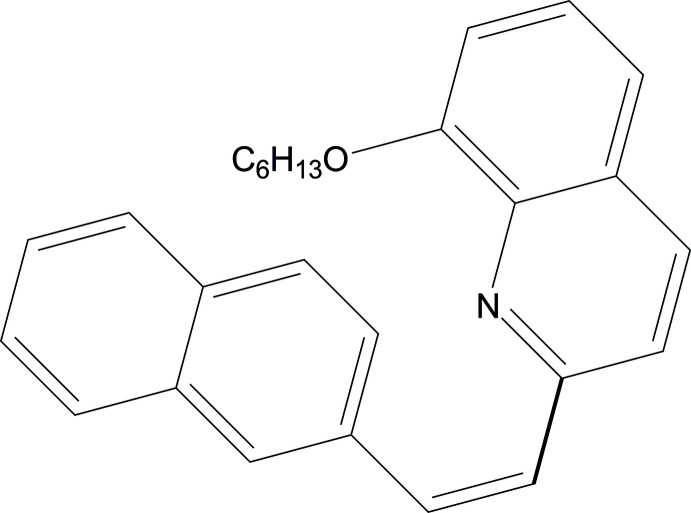


## 2. Structural commentary

**Figure 1:**
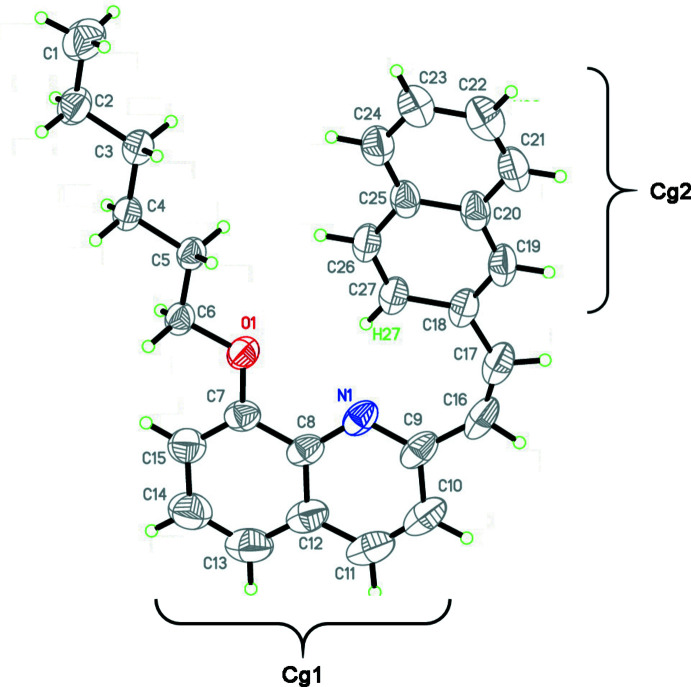
Mol­ecular structure of compound (**1**) with atom labelling. Atomic displacement ellipsoids are drawn at the 30% probability level.

Compound (**1**) crystallizes in the monoclinic centrosymmetric space group *P*2_1_/*n* with one mol­ecule per asymmetric unit (Fig. 1 ▸). The mol­ecule contains four fragments, which are planar within experimental error, *viz*. the quinoline (*Cg*1) and naphthalene (*Cg*2) systems, the C9—C16=C17—C18 bridge and the hex­yloxy chain, which adopts an all-*trans* conformation. Planes *Cg*1 and *Cg*2 subtend a dihedral angle of 15.46 (5)°, and angles of 8.34 (8) and 13.28 (10)°, respectively, with the bridge plane. The *Cg*1 and the hex­yloxy planes form an angle of 5.05 (4)°. Thus, all non-hydrogen atoms in the mol­ecule are roughly coplanar, with an r.m.s. deviation of 0.23 Å. The intra­molecular (transannular) contact C27—H27⋯N1 [C⋯N = 3.068 (3), C—H = 0.955 (16), H⋯N = 2.195 (16) Å, C—H⋯N = 151.4 (13)°; Table 1 ▸], is much shorter than the standard van der Waals contacts C⋯N (3.31 Å) and H⋯N (2.59 Å) (Rowland & Taylor, 1996 ▸) and has a deceptive appearance of a rather strong intra­molecular hydrogen bond (Desiraju & Steiner, 1999 ▸). However, the bond angles in the vinyl bridge, C9—C16=C17 of 136.9 (2)° and C16=C17—C18 of 137.4 (2)°, are much wider than in non-planar pyridyl-vinyl-benzene moieties without C—H⋯N inter­actions (see *Database survey*), indicating that the C27—H27⋯N1 contact may in fact be a repulsive, ‘collateral damage’ type contact (Gavezzotti, 2010 ▸) and the bridge absorbs the resulting strain. On the contrary, the geometry of the longer transannular contact C27—H27⋯O1 [C⋯O = 3.633 (2), H⋯O = 2.809 (17) Å, C—H⋯O = 145.0 (12)°] corresponds to that of a weakly stabilizing hydrogen bond (Steiner, 1996 ▸; Desiraju & Steiner, 1999 ▸).

## 3. Supra­molecular features

**Figure 2:**
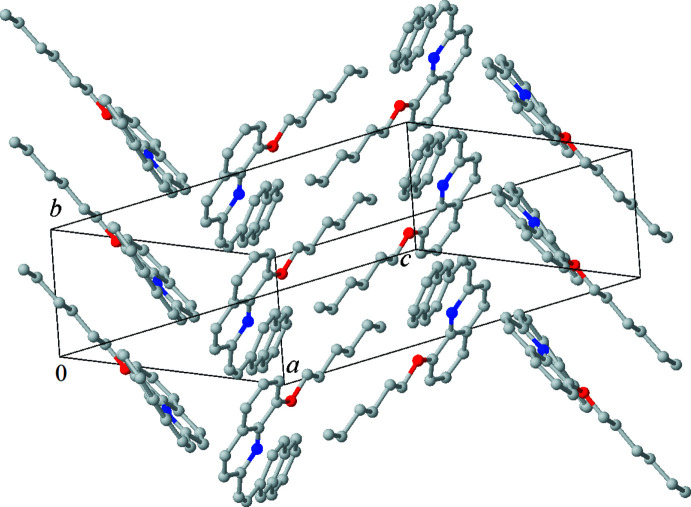
Crystal packing of compound (**1**). Hydrogen atoms are omitted for clarity.

In the crystal, mol­ecules related by *b* translation pack face-to-face, forming strongly slanted stacks running along the *b-*axis direction (see Fig. 2 ▸). However, π–π stacking between *aromatic* moieties (Hunter & Sanders, 1990 ▸) is practically absent. Thus, although the quinoline (*Cg*1) π-systems are parallel, their overlap is marginal, involving only one carbon atom on either side, with a C10⋯C15(*x*, *y* + 1, *z*) contact distance of 3.540 (3) Å, while the naphthalene moiety overlaps with the alkyl chain of the next mol­ecule. Mol­ecules belonging to different stacks and related by a screw axis form a typical γ-motif (Loots & Barbour, 2012 ▸), their quinoline and naphthalene moieties contact at an inter­planar angle of 68.60 (5)°. The packing of the *n*-hexyl chains resembles that of pure alkanes, with a parallel arrangement of the chains, which adopt an all-*trans* conformation.

## 4. Database survey

The Cambridge Crystallographic Database (CSD Version 5.42, November 2021; Groom *et al.*, 2016 ▸) contains only two structures with a 2-[2-(naphthalen-2-yl)ethen­yl]quinoline moiety, *viz*. bis­{μ-2-[2-(2-naphth­yl)vin­yl]quinolin-8-olato}bis­(di­meth­yl sulfoxide)­bis­(iodo)­dicadmium(II) (GAQFIQ; Yuan *et al.*, 2017 ▸) and 2-[2-(6-meth­oxy­naphthalen-2-yl)vin­yl]-1-methyl­quinolin-1-ium iodide (LEWXAP; Tian *et al.*, 2018 ▸). Both have a *trans*-configuration about the vinyl C=C bond, in contrast with the *cis*-configuration of mol­ecule (**1**), and adopt more planar conformation than the latter. In *cis*-2,5-bis­(2-methyl­but­oxy)- and *cis*-2,5-dibut­oxy-1,4-bis­[2-(pyrid-2-yl)vin­yl]benzene (SIXQOH and SIXQUN; Liu *et al.*, 2014 ▸), the (pyrid-2-yl)vinyl­benzene fragments have a *cis*-configuration about the C=C bond, but in both structures the pyridyl N atom is oriented outward, not intra­annularly. Thus the pyridyl and benzene rings cannot be coplanar with the ethenyl bridge, due to the steric repulsion between *ortho*-H atoms, and are inclined to this bridge by 28–47° in a propeller-like fashion. The C—C=C bond angles in the vinyl bridge (129–131°) are narrower than in (**1**).

## 5. Synthesis and crystallization

**Figure 3:**
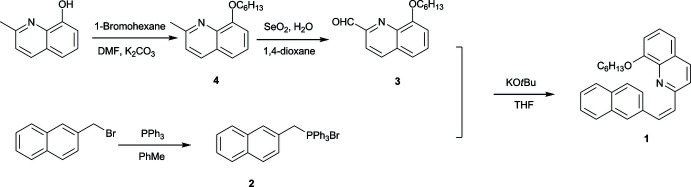
Synthetic procedures for (*Z*)-8-(hex­yloxy)-2-[2-(naphthalen-2-yl)ethen­yl]quinoline (**1**).

All reactants and solvents were purchased and used without further purification. THF was dried by using Na in the presence of benzo­phenone. Bromo­(naphthalen-2-ylmeth­yl)tri­phenyl­phospho­rane (**2**) was synthesized according to the literature method of our research group (Luo *et al.*, 2018 ▸). ^1^H NMR and ^13^C NMR spectra were obtained in CDCl_3_ with tetra­methyl­silane as inter­nal standard on a Bruker Advance spectrometer. HRMS spectra were obtained on a 650Q-TOF spectrograph (Agilent). The synthesis procedures for compounds (**1**)–(**4**) are shown in Fig. 3 ▸.

8-(Hex­yloxy)-2-methyl­quinoline (**4**): 8-hy­droxy-2-methyl-quinoline (477 mg, 3.0 mmol), 1-bromo­hexane (495 mg, 3.0 mmol), K_2_CO_3_ (207 mg, 1.5 mmol) and DMF (10 ml) were mixed in a flask and stirred for 16 h at room temperature. Then the organic phase was extracted with di­chloro­methane and water. After the solvent had been removed under reduced pressure, the residue was purified by flash chromatography on silica gel using ethyl acetate–petroleum ether (3:50) as the eluent to obtain a white grease (495 mg). Yield: 67.8%. ^1^H NMR (300 MHz, CDCl_3_, δ) 7.99 (*d*, *J* = 8.4 Hz, 1H), 7.40–7.26 (*m*, 3H), 7.03 (*dd*, *J* = 7.2 Hz, 1H), 4.23 (*t*, *J* = 7.2 Hz, 2H), 2.78 (*s*, 3H), 2.08–1.98 (*m*, 2H), 1.54–1.47 (*m*, 2H), 1.43–1.33 (*m*, 4H), 0.98–0.89 (*m*, 3H). ^13^C NMR (400 MHz, CDCl_3_, δ) 157.97, 154.37, 139.97, 135.99, 127.69, 125.64, 122.37, 119.23, 109.01, 69.13, 31.66, 28.82, 25.72, 25.68, 22.60, 14.02.

8-(Hex­yloxy)quinoline-2-carbaldehyde (**3**): Compound (**4**) (3 g, 12.3 mmol), SeO_2_ (1.74 g, 15.7 mmol) and 1,4-dioxane (300 ml) were mixed in a three-necked flask, heated to 368 K and stirred at this temperature for 24 h. The reaction solution was extracted with di­chloro­methane and water. After the solvent had been removed under reduced pressure, the residue was purified by flash chromatography on silica gel using ethyl acetate–petroleum ether (2:25) as the eluent, to obtain a yellow solid (1.63 g). Yield: 51.5%. ^1^H NMR (300 MHz, CDCl_3_, δ) 10.29 (*d*, *J* = 0.9 Hz, 1H), 8.26 (*d*, *J* = 8.4 Hz, 1H), 8.05 (*d*, *J* = 8.4 Hz, 1H), 7.60 (*t*, *J* = 8.1 Hz, 1H), 7.45–7.42 (*m*, 1H), 7.16–7.13 (*m*, 1H), 4.29 (*t*, *J* = 6.9 Hz, 2H), 2.11–2.01 (*m*, 2H), 1.62–1.53 (*m*, 2H), 1.47–1.35 (*m*, 4H), 0.95–0.90 (*m*, 3H). ^13^C NMR (400 MHz, CDCl_3_, d) 193.87, 155.68, 151.40, 140.15, 137.20, 131.39, 129.80, 119.33, 117.74, 109.69, 69.44, 31.61, 28.85, 25.69, 22.59, 14.03.

(*Z*)-8-(Hex­yloxy)-2-[2-(naphthalen-2-yl)ethen­yl]quinoline (**1**): bromo­(naphthalen-2-ylmeth­yl)tri­phenyl­phospho­rane (**2**) (2250 mg, 4.6 mmol) was dissolved in anhydrous tetra­hydro­furan (10 mL) under Ar and the solution was cooled to 273 K. KO*t*Bu (1000 mg, 8.9 mmol) was added and stirred for 15 min. A solution of compound (**3**) (1290 mg, 5.0 mmol) in dry THF (10 mL) was added dropwise into the reaction mixture. After the addition, the mixture was stirred for 15 min. A few drops of water were added to quench the reaction. The mixture was extracted with CH_2_Cl_2_. The organic layer was washed with water three times and dried over anhydrous Na_2_SO_4_. The solvent was removed *in vacuo* and the residue was purified by flash chromatography on silica gel using di­chloro­methane–petroleum ether (1:10) as the eluent to afford a white solid (1500mg). Yield: 84.6%. Slow evaporation of compound (**1**) from di­chloro­methane/ethanol mixed solutions yielded light-yellow block-shaped crystals of (**1**). ^1^H NMR (300 MHz, CDCl_3_, δ) 8.08 (*d*, *J* = 8Hz, 1H), 7.97 (*s*, 1H), 7.85–7.80 (*m*, 5H), 7.73 (*d*, *J* = 8 Hz, 1H), 7.62–7.58 (*m*, 1H), 7.49–7.46 (*m*, 2H), 7.41–7.33 (*m*, 2H), 7.06 (*d*, *J* = 8 Hz, 1H), 4.26 (*t*, *J* = 8 Hz, 2H), 2.10–2.07 (*m*, 2H), 1.60–1.58 (*m*, 2H), 1.45–1.42 (*m*, 4H), 0.97–0.93 (*m*, 3H). ^13^C NMR (400 MHz, CDCl_3_, δ) 154.88, 140.44, 136.25, 134.33, 133.80, 133.65, 133.47, 130.11, 128.56, 128.45, 128.25, 127.92, 127.75, 126.40, 126.38, 126.29, 123.79, 119.47, 119.29, 109.41, 69.30, 31.75, 28.99, 25.81, 22.70, 14.12. HRMS (*m*/*z*): 382.2169 [*M* + H]^+^ (calculated for C_27_H_27_NO: 382.2126).

## 6. Refinement

Crystal data, data collection and structure refinement details are summarized in Table 2 ▸. C-bound H atoms were refined using a riding model with C—H = 0.93–0.97 Å and *U*_iso_(H) = 1.2–1.5*U*_eq_(C), except for H27, which was refined in an isotropic approximation.

## Supplementary Material

Crystal structure: contains datablock(s) I. DOI: 10.1107/S2056989022006740/zv2017sup1.cif

Structure factors: contains datablock(s) I. DOI: 10.1107/S2056989022006740/zv2017Isup2.hkl

Word document (1. Synthesis Procedures 2. Date of short ring-interactions 3. NMR, HRMS Spectra ). DOI: 10.1107/S2056989022006740/zv2017sup5.docx

Supporting information file. DOI: 10.1107/S2056989022006740/zv2017Isup4.cml

CCDC reference: 2182952

Additional supporting information:  crystallographic information; 3D view; checkCIF report

## Figures and Tables

**Table 1:** Hydrogen-bond geometry (Å, °)

*D*—H⋯*A*	*D*—H	H⋯*A*	*D*⋯*A*	*D*—H⋯*A*
C6—H6*A*⋯N1^i^	0.97	2.68	3.600 (2)	159
C27—H27⋯O1	0.955 (16)	2.809 (17)	3.633 (2)	145.0 (12)
C27—H27⋯N1	0.955 (16)	2.195 (16)	3.068 (3)	151.4 (13)

Symmetry code: (i) 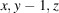 .

**Table 2:** Experimental details

Crystal data	
Chemical formula	C_27_H_27_NO
*M*_r_	381.49
Crystal system, space group	Monoclinic, *P*2_1_/*n*
Temperature (K)	293
*a*, *b*, *c* (Å)	14.416 (3), 5.8569 (10), 25.354 (5)
β (°)	96.116 (3)
*V* (Å^3^)	2128.6 (7)
*Z*	4
Radiation type	Mo *K*α
μ (mm^−1^)	0.07
Crystal size (mm)	0.20 × 0.19 × 0.13

Data collection	
Diffractometer	Bruker APEXIII CCD
Absorption correction	Multi-scan (*SADABS*; Bruker, 2017 ▸)
*T*_min_, *T*_max_	0.667, 0.746
No. of measured, independent and observed [*I* > 2σ(*I*)] reflections	23828, 4844, 2314
*R*_int_	0.041
(sin θ/λ)_max_ (Å^−1^)	0.648

Refinement	
*R*[*F* ^2^ > 2σ(*F* ^2^)], *wR*(*F* ^2^), *S*	0.045, 0.140, 1.00
No. of reflections	4844
No. of parameters	268
H-atom treatment	H atoms treated by a mixture of independent and constrained refinement
Δρ_max_, Δρ_min_ (e Å^−3^)	0.12, −0.11

Computer programs: *APEX3* and *SAINT* (Bruker, 2017 ▸), *SHELXT2014/4* (Sheldrick, 2015*a* ▸), *SHELXL2018/3* (Sheldrick, 2015*b* ▸), *ORTEP-3 for Windows* (Farrugia, 2012 ▸) and *PLATON* (Spek, 2020 ▸).

## supplementary crystallographic information

### Crystal data

**Table:**

C_27_H_27_NO	*F*(000) = 816
*M**_r_* = 381.49	*D*_x_ = 1.190 Mg m^−^^3^
Monoclinic, *P*2_1_/*n*	Mo *K*α radiation, λ = 0.71073 Å
*a* = 14.416 (3) Å	Cell parameters from 2641 reflections
*b* = 5.8569 (10) Å	θ = 2.7–19.7°
*c* = 25.354 (5) Å	µ = 0.07 mm^−^^1^
β = 96.116 (3)°	*T* = 293 K
*V* = 2128.6 (7) Å^3^	Block, light yellow
*Z* = 4	0.20 × 0.19 × 0.13 mm

### Data collection

**Table:**

Bruker APEXIII CCD diffractometer	4844 independent reflections
Radiation source: fine-focus sealed tube	2314 reflections with *I* > 2σ(*I*)
Graphite monochromator	*R*_int_ = 0.041
φ and ω scans	θ_max_ = 27.4°, θ_min_ = 1.6°
Absorption correction: multi-scan (SADABS; Bruker, 2017)	*h* = −18→18
*T*_min_ = 0.667, *T*_max_ = 0.746	*k* = −7→7
23828 measured reflections	*l* = −32→32

### Refinement

**Table:**

Refinement on *F*^2^	Secondary atom site location: difference Fourier map
Least-squares matrix: full	Hydrogen site location: inferred from neighbouring sites
*R*[*F*^2^ > 2σ(*F*^2^)] = 0.045	H atoms treated by a mixture of independent and constrained refinement
*wR*(*F*^2^) = 0.140	*w* = 1/[σ^2^(*F*_o_^2^) + (0.0588*P*)^2^ + 0.0643*P*] where *P* = (*F*_o_^2^ + 2*F*_c_^2^)/3
*S* = 1.00	(Δ/σ)_max_ = 0.001
4844 reflections	Δρ_max_ = 0.12 e Å^−^^3^
268 parameters	Δρ_min_ = −0.11 e Å^−^^3^
0 restraints	Extinction correction: SHELXL-2018/3 (Sheldrick, 2015b), Fc^*^=kFc[1+0.001xFc^2^λ^3^/sin(2θ)]^-1/4^
Primary atom site location: dual	Extinction coefficient: 0.0047 (10)

### Special details

**Table:**

Geometry. All esds (except the esd in the dihedral angle between two l.s. planes) are estimated using the full covariance matrix. The cell esds are taken into account individually in the estimation of esds in distances, angles and torsion angles; correlations between esds in cell parameters are only used when they are defined by crystal symmetry. An approximate (isotropic) treatment of cell esds is used for estimating esds involving l.s. planes.
Refinement. Refinement of F^2^ against ALL reflections. The weighted R-factor wR and goodness of fit S are based on F^2^, conventional R-factors R are based on F, with F set to zero for negative F^2^. The threshold expression of F^2^ > 2sigma(F^2^) is used only for calculating R-factors(gt) etc. and is not relevant to the choice of reflections for refinement. R-factors based on F^2^ are statistically about twice as large as those based on F, and R- factors based on ALL data will be even larger.

### Fractional atomic coordinates and isotropic or equivalent isotropic displacement parameters (Å^2^)

**Table:**

	*x*	*y*	*z*	*U*_iso_*/*U*_eq_	
O1	0.66156 (7)	0.62694 (18)	0.57990 (4)	0.0757 (3)	
N1	0.75177 (10)	0.9822 (2)	0.62699 (6)	0.0781 (4)	
C1	0.22807 (16)	−0.0537 (4)	0.58497 (10)	0.1328 (8)	
H1A	0.237845	−0.066613	0.622896	0.199*	
H1B	0.196935	−0.188067	0.570409	0.199*	
H1C	0.190330	0.078059	0.575470	0.199*	
C2	0.31962 (13)	−0.0297 (3)	0.56346 (8)	0.0949 (6)	
H2A	0.308734	−0.016894	0.525157	0.114*	
H2B	0.355594	−0.167615	0.571548	0.114*	
C3	0.37715 (11)	0.1737 (3)	0.58472 (7)	0.0776 (5)	
H3A	0.341148	0.311844	0.576951	0.093*	
H3B	0.388916	0.160265	0.622982	0.093*	
C4	0.46867 (11)	0.1963 (2)	0.56210 (6)	0.0700 (4)	
H4A	0.457115	0.204372	0.523752	0.084*	
H4B	0.505672	0.060613	0.570984	0.084*	
C5	0.52431 (10)	0.4048 (3)	0.58210 (6)	0.0672 (4)	
H5A	0.487190	0.540674	0.573657	0.081*	
H5B	0.536989	0.395793	0.620387	0.081*	
C6	0.61429 (10)	0.4259 (3)	0.55851 (6)	0.0700 (4)	
H6A	0.652577	0.292010	0.567038	0.084*	
H6B	0.602656	0.437927	0.520220	0.084*	
C7	0.74858 (12)	0.6676 (3)	0.56615 (7)	0.0764 (5)	
C8	0.79621 (12)	0.8568 (3)	0.59206 (7)	0.0771 (5)	
C9	0.79590 (14)	1.1602 (3)	0.65059 (8)	0.0875 (6)	
C16	0.75188 (17)	1.3102 (3)	0.68707 (9)	0.0982 (7)	
H16	0.785723	1.444059	0.693853	0.118*	
C17	0.67630 (17)	1.3052 (3)	0.71303 (8)	0.0955 (6)	
H17	0.671267	1.437542	0.732806	0.115*	
C18	0.59923 (13)	1.1490 (3)	0.71893 (7)	0.0795 (5)	
C19	0.53998 (16)	1.2047 (3)	0.75602 (8)	0.0898 (6)	
H19	0.549977	1.340765	0.774655	0.108*	
C20	0.46487 (15)	1.0652 (3)	0.76708 (7)	0.0852 (5)	
C25	0.44875 (13)	0.8617 (3)	0.73757 (7)	0.0781 (5)	
C24	0.37471 (15)	0.7199 (3)	0.74859 (8)	0.0943 (6)	
H24	0.362938	0.586709	0.729063	0.113*	
C23	0.32015 (16)	0.7732 (4)	0.78701 (9)	0.1080 (7)	
H23	0.271208	0.677419	0.793551	0.130*	
C10	0.88783 (17)	1.2183 (4)	0.64027 (10)	0.1111 (8)	
H10	0.917581	1.343370	0.657155	0.133*	
C11	0.93200 (16)	1.0934 (5)	0.60626 (12)	0.1162 (8)	
H11	0.992619	1.131267	0.600205	0.139*	
C12	0.88748 (14)	0.9054 (4)	0.57959 (9)	0.0966 (6)	
C13	0.92800 (16)	0.7724 (5)	0.54240 (12)	0.1219 (8)	
H13	0.987830	0.805954	0.534141	0.146*	
C14	0.88038 (17)	0.5945 (5)	0.51830 (10)	0.1216 (8)	
H14	0.907909	0.506682	0.493677	0.146*	
C15	0.79016 (13)	0.5420 (3)	0.53017 (8)	0.0948 (6)	
H15	0.758316	0.419785	0.513260	0.114*	
C21	0.40711 (18)	1.1152 (4)	0.80704 (9)	0.1059 (7)	
H21	0.417029	1.248223	0.826883	0.127*	
C22	0.33727 (18)	0.9717 (5)	0.81684 (9)	0.1175 (7)	
H22	0.300382	1.005901	0.843723	0.141*	
C26	0.50926 (13)	0.8091 (3)	0.69930 (7)	0.0820 (5)	
H26	0.498761	0.675917	0.679590	0.098*	
C27	0.58201 (15)	0.9433 (3)	0.68985 (8)	0.0783 (5)	
H27	0.6259 (11)	0.906 (3)	0.6655 (6)	0.078 (5)*	

### Atomic displacement parameters (Å^2^)

**Table:**

	*U*^11^	*U*^22^	*U*^33^	*U*^12^	*U*^13^	*U*^23^
O1	0.0716 (7)	0.0719 (7)	0.0811 (7)	−0.0082 (6)	−0.0033 (6)	−0.0069 (6)
N1	0.0824 (10)	0.0672 (9)	0.0775 (9)	−0.0120 (8)	−0.0253 (8)	0.0134 (8)
C1	0.1192 (17)	0.1313 (19)	0.153 (2)	−0.0422 (15)	0.0367 (15)	−0.0115 (16)
C2	0.0978 (14)	0.0809 (12)	0.1056 (15)	−0.0184 (11)	0.0093 (11)	−0.0081 (11)
C3	0.0849 (12)	0.0691 (11)	0.0775 (11)	−0.0055 (9)	0.0029 (9)	−0.0039 (9)
C4	0.0801 (11)	0.0598 (10)	0.0680 (10)	−0.0002 (8)	−0.0028 (8)	−0.0060 (8)
C5	0.0724 (10)	0.0637 (10)	0.0628 (9)	0.0000 (8)	−0.0062 (8)	−0.0066 (8)
C6	0.0739 (11)	0.0623 (10)	0.0699 (10)	0.0014 (8)	−0.0101 (8)	−0.0044 (8)
C7	0.0658 (11)	0.0789 (12)	0.0820 (12)	0.0020 (10)	−0.0041 (9)	0.0127 (10)
C8	0.0677 (11)	0.0755 (12)	0.0823 (12)	−0.0057 (10)	−0.0185 (9)	0.0214 (10)
C9	0.0927 (14)	0.0760 (13)	0.0842 (13)	−0.0220 (11)	−0.0344 (10)	0.0202 (11)
C16	0.1201 (17)	0.0702 (13)	0.0933 (15)	−0.0284 (13)	−0.0399 (13)	0.0032 (12)
C17	0.1251 (17)	0.0646 (12)	0.0876 (14)	−0.0103 (12)	−0.0314 (13)	−0.0030 (10)
C18	0.1019 (13)	0.0532 (10)	0.0750 (12)	0.0031 (10)	−0.0297 (10)	0.0013 (9)
C19	0.1233 (16)	0.0561 (11)	0.0825 (13)	0.0169 (12)	−0.0246 (12)	−0.0110 (10)
C20	0.1076 (15)	0.0621 (11)	0.0804 (12)	0.0226 (11)	−0.0151 (11)	−0.0041 (10)
C25	0.0958 (13)	0.0607 (11)	0.0734 (11)	0.0165 (10)	−0.0107 (10)	−0.0039 (9)
C24	0.1070 (15)	0.0751 (13)	0.0999 (15)	0.0079 (12)	0.0072 (12)	−0.0066 (11)
C23	0.1192 (17)	0.0947 (15)	0.1114 (17)	0.0172 (13)	0.0181 (14)	−0.0045 (13)
C10	0.1001 (17)	0.1007 (17)	0.1222 (19)	−0.0364 (14)	−0.0362 (14)	0.0227 (14)
C11	0.0784 (14)	0.119 (2)	0.144 (2)	−0.0257 (14)	−0.0191 (14)	0.0392 (17)
C12	0.0756 (13)	0.0937 (15)	0.1155 (16)	−0.0120 (12)	−0.0124 (12)	0.0281 (13)
C13	0.0795 (15)	0.130 (2)	0.158 (2)	−0.0036 (15)	0.0198 (15)	0.0251 (18)
C14	0.0943 (16)	0.125 (2)	0.149 (2)	0.0034 (15)	0.0317 (15)	0.0036 (17)
C15	0.0820 (14)	0.0948 (14)	0.1071 (15)	0.0011 (11)	0.0069 (11)	0.0023 (12)
C21	0.1363 (19)	0.0830 (14)	0.0953 (15)	0.0325 (14)	−0.0017 (14)	−0.0196 (12)
C22	0.136 (2)	0.1138 (19)	0.1052 (17)	0.0295 (16)	0.0217 (14)	−0.0059 (15)
C26	0.1007 (13)	0.0613 (11)	0.0811 (12)	−0.0008 (10)	−0.0045 (10)	−0.0134 (9)
C27	0.0940 (14)	0.0608 (11)	0.0761 (12)	−0.0013 (10)	−0.0101 (10)	−0.0066 (9)

### Geometric parameters (Å, º)

**Table:**

O1—C6	1.4374 (17)	C17—C18	1.459 (3)
O1—C7	1.3582 (19)	C18—C19	1.375 (2)
N1—C8	1.362 (2)	C18—C27	1.420 (2)
N1—C9	1.330 (2)	C19—H19	0.9300
C1—H1A	0.9600	C19—C20	1.408 (3)
C1—H1B	0.9600	C20—C25	1.413 (2)
C1—H1C	0.9600	C20—C21	1.409 (3)
C1—C2	1.487 (3)	C25—C24	1.404 (2)
C2—H2A	0.9700	C25—C26	1.406 (2)
C2—H2B	0.9700	C24—H24	0.9300
C2—C3	1.517 (2)	C24—C23	1.352 (3)
C3—H3A	0.9700	C23—H23	0.9300
C3—H3B	0.9700	C23—C22	1.395 (3)
C3—C4	1.500 (2)	C10—H10	0.9300
C4—H4A	0.9700	C10—C11	1.342 (3)
C4—H4B	0.9700	C11—H11	0.9300
C4—C5	1.518 (2)	C11—C12	1.411 (3)
C5—H5A	0.9700	C12—C13	1.398 (3)
C5—H5B	0.9700	C13—H13	0.9300
C5—C6	1.490 (2)	C13—C14	1.357 (3)
C6—H6A	0.9700	C14—H14	0.9300
C6—H6B	0.9700	C14—C15	1.400 (3)
C7—C8	1.427 (2)	C15—H15	0.9300
C7—C15	1.360 (2)	C21—H21	0.9300
C8—C12	1.415 (3)	C21—C22	1.355 (3)
C9—C16	1.468 (3)	C22—H22	0.9300
C9—C10	1.419 (3)	C26—H26	0.9300
C16—H16	0.9300	C26—C27	1.352 (2)
C16—C17	1.332 (3)	C27—H27	0.955 (16)
C17—H17	0.9300		
			
C7—O1—C6	117.42 (13)	C16—C17—C18	137.4 (2)
C9—N1—C8	118.67 (17)	C18—C17—H17	111.3
H1A—C1—H1B	109.5	C19—C18—C17	117.15 (18)
H1A—C1—H1C	109.5	C19—C18—C27	117.99 (19)
H1B—C1—H1C	109.5	C27—C18—C17	124.9 (2)
C2—C1—H1A	109.5	C18—C19—H19	118.5
C2—C1—H1B	109.5	C18—C19—C20	123.04 (17)
C2—C1—H1C	109.5	C20—C19—H19	118.5
C1—C2—H2A	108.6	C19—C20—C25	118.06 (19)
C1—C2—H2B	108.6	C19—C20—C21	123.2 (2)
C1—C2—C3	114.63 (16)	C21—C20—C25	118.7 (2)
H2A—C2—H2B	107.6	C24—C25—C20	118.62 (19)
C3—C2—H2A	108.6	C24—C25—C26	123.26 (17)
C3—C2—H2B	108.6	C26—C25—C20	118.10 (19)
C2—C3—H3A	108.8	C25—C24—H24	119.3
C2—C3—H3B	108.8	C23—C24—C25	121.3 (2)
H3A—C3—H3B	107.7	C23—C24—H24	119.3
C4—C3—C2	113.78 (14)	C24—C23—H23	120.0
C4—C3—H3A	108.8	C24—C23—C22	120.0 (2)
C4—C3—H3B	108.8	C22—C23—H23	120.0
C3—C4—H4A	108.9	C9—C10—H10	119.9
C3—C4—H4B	108.9	C11—C10—C9	120.3 (2)
C3—C4—C5	113.47 (13)	C11—C10—H10	119.9
H4A—C4—H4B	107.7	C10—C11—H11	119.6
C5—C4—H4A	108.9	C10—C11—C12	120.7 (2)
C5—C4—H4B	108.9	C12—C11—H11	119.6
C4—C5—H5A	109.1	C11—C12—C8	115.9 (2)
C4—C5—H5B	109.1	C13—C12—C8	120.3 (2)
H5A—C5—H5B	107.8	C13—C12—C11	123.8 (2)
C6—C5—C4	112.59 (13)	C12—C13—H13	119.9
C6—C5—H5A	109.1	C14—C13—C12	120.2 (2)
C6—C5—H5B	109.1	C14—C13—H13	119.9
O1—C6—C5	108.33 (12)	C13—C14—H14	119.7
O1—C6—H6A	110.0	C13—C14—C15	120.6 (2)
O1—C6—H6B	110.0	C15—C14—H14	119.7
C5—C6—H6A	110.0	C7—C15—C14	120.8 (2)
C5—C6—H6B	110.0	C7—C15—H15	119.6
H6A—C6—H6B	108.4	C14—C15—H15	119.6
O1—C7—C8	115.49 (17)	C20—C21—H21	119.7
O1—C7—C15	124.23 (17)	C22—C21—C20	120.7 (2)
C15—C7—C8	120.28 (18)	C22—C21—H21	119.7
N1—C8—C7	118.79 (16)	C23—C22—H22	119.7
N1—C8—C12	123.38 (19)	C21—C22—C23	120.7 (2)
C12—C8—C7	117.8 (2)	C21—C22—H22	119.7
N1—C9—C16	122.30 (18)	C25—C26—H26	118.5
N1—C9—C10	121.1 (2)	C27—C26—C25	123.01 (17)
C10—C9—C16	116.6 (2)	C27—C26—H26	118.5
C9—C16—H16	111.6	C18—C27—H27	115.8 (10)
C17—C16—C9	136.87 (19)	C26—C27—C18	119.8 (2)
C17—C16—H16	111.6	C26—C27—H27	124.3 (10)
C16—C17—H17	111.3		
			
O1—C7—C8—N1	−1.1 (2)	C17—C18—C27—C26	178.78 (16)
O1—C7—C8—C12	179.39 (14)	C18—C19—C20—C25	−1.6 (2)
O1—C7—C15—C14	−179.77 (16)	C18—C19—C20—C21	176.41 (16)
N1—C8—C12—C11	0.8 (2)	C19—C18—C27—C26	−0.2 (2)
N1—C8—C12—C13	−178.52 (17)	C19—C20—C25—C24	179.35 (15)
N1—C9—C16—C17	13.5 (3)	C19—C20—C25—C26	0.7 (2)
N1—C9—C10—C11	0.0 (3)	C19—C20—C21—C22	−178.30 (18)
C1—C2—C3—C4	−179.36 (17)	C20—C25—C24—C23	−0.9 (3)
C2—C3—C4—C5	177.97 (13)	C20—C25—C26—C27	0.4 (2)
C3—C4—C5—C6	−179.12 (13)	C20—C21—C22—C23	−1.0 (3)
C4—C5—C6—O1	−179.87 (11)	C25—C20—C21—C22	−0.3 (3)
C6—O1—C7—C8	−174.45 (12)	C25—C24—C23—C22	−0.4 (3)
C6—O1—C7—C15	5.9 (2)	C25—C26—C27—C18	−0.7 (3)
C7—O1—C6—C5	175.35 (12)	C24—C25—C26—C27	−178.17 (16)
C7—C8—C12—C11	−179.80 (15)	C24—C23—C22—C21	1.4 (3)
C7—C8—C12—C13	0.9 (3)	C10—C9—C16—C17	−168.3 (2)
C8—N1—C9—C16	177.45 (15)	C10—C11—C12—C8	−1.4 (3)
C8—N1—C9—C10	−0.7 (2)	C10—C11—C12—C13	177.8 (2)
C8—C7—C15—C14	0.6 (3)	C11—C12—C13—C14	−179.8 (2)
C8—C12—C13—C14	−0.5 (3)	C12—C13—C14—C15	0.2 (4)
C9—N1—C8—C7	−179.17 (13)	C13—C14—C15—C7	−0.2 (3)
C9—N1—C8—C12	0.3 (2)	C15—C7—C8—N1	178.53 (15)
C9—C16—C17—C18	0.6 (4)	C15—C7—C8—C12	−0.9 (2)
C9—C10—C11—C12	1.1 (3)	C21—C20—C25—C24	1.2 (2)
C16—C9—C10—C11	−178.2 (2)	C21—C20—C25—C26	−177.45 (15)
C16—C17—C18—C19	172.5 (2)	C26—C25—C24—C23	177.68 (17)
C16—C17—C18—C27	−6.5 (3)	C27—C18—C19—C20	1.4 (2)
C17—C18—C19—C20	−177.69 (15)		

### Hydrogen-bond geometry (Å, º)

**Table:**

*D*—H···*A*	*D*—H	H···*A*	*D*···*A*	*D*—H···*A*
C6—H6*A*···N1^i^	0.97	2.68	3.600 (2)	159
C27—H27···O1	0.955 (16)	2.809 (17)	3.633 (2)	145.0 (12)
C27—H27···N1	0.955 (16)	2.195 (16)	3.068 (3)	151.4 (13)

Symmetry code: (i) *x*, *y*−1, *z*.
