# Supplementary material for: Crystal structure of 8-hex­yloxy-2-[(Z)-2-(naph­thal­en-2-yl)ethen­yl]quinoline
Source: Acta Crystallogr E Crystallogr Commun. 2022 Jul 5;78(Pt 8):770–3. doi: 10.1107/S2056989022006740 (PMC9361378; doi:10.1107/S2056989022006740)
Supplement: Supplementary file 3 [file e-78-00770-sup5.docx]

**Supporting Information**

**Table of contents**

1. Synthesis Procedures

2. NMR, HRMS Spectra

**1. Synthesis Procedures**

**8-(hexyloxy)-2-methylquinoline (4):** The 8-hydroxy-2-methyl-quinoline (477 mg, 3 mmol), 1-Bromohexane (495 mg, 3 mmol), K_2_CO_3_ (207 mg) and DMF (10 ml) were mixed to the flask and stirred for 16 h under room temperature. Then, the organic phase was extracted with dichloromethane and water. After the solvent was removed under reduced pressure, the residuum was purified by flash chromatography on silica gel using ethyl acetate-petroleum ether (3: 50) as the eluent to achieve white grease (495 mg). Yield: 87%. ^1^H NMR (300 MHz, CDCl_3_, δ) 7.99 ( d , *J* = 8.4 Hz, 1H), 7.40-7.26 (m, 3H), 7.03 (dd, *J* = 7.2 Hz, 1H), 4.23 (t, *J* = 7.2 Hz, 2H), 2.78 (s, 3H), 2.08-1.98 (m, 2H), 1.54-1.47 (m, 2H), 1.43-1.33 (m, 4H),0.98-0.89(m, 3H). ^13^C NMR (300 MHz, CDCl_3_, δ) 157.97, 154.37, 139.97, 135.99, 127.69, 125.64, 122.37, 119.23, 109.01, 69.13, 31.66, 28.82, 25.72, 25.68, 22.60, 14.02.

**8-(hexyloxy)quinoline-2-carbaldehyde (3):** Compound **4** (3 g, 12.4 mmol), SeO_2_ (1.74 g, 15.8 mmol) and 1,4-dioxane（300 ml）were mixed to the three necked flask, heated to 95°C and stirred at this temperature for 24 h. The reaction solution was extracted with dichloromethane and water. After the solvent was removed under reduced pressure, the residuum was purified by flash chromatography on silica gel using ethyl acetate-petroleum ether (2: 25) as the eluent to achieve a yellow solid (1.63 g). Yield: 78%. ^1^H NMR (300 MHz, CDCl3, δ) 10.29 (d, *J* = 0.9 Hz, 1H ), 8.26 (d, *J* = 8.4 Hz, 1H), 8.05(d, *J* = 8.4 Hz, 1H), 7.60 (t, *J* = 8.1 Hz, 1H), 7.45-7.42(m, 1H), 7.16-7.13 (m, 1H), 4.29(t, *J* = 6.9Hz, 2H), 2.11-2.01(m, 2H), 1.62-1.53(m, 2H), 1.47-1.35(m,4H), 0.95-0.90(m, 3H). ^13^C NMR (300 MHz, CDCl_3_, δ) 193.87, 155.68, 151.40, 140.15, 137.20, 131.39, 129.80, 119.33, 117.74, 109.69, 69.44, 31.61, 28.85, 25.69, 22.59, 14.03.

**(*Z*)-8-(hexyloxy)-2-(2-(naphthalen-2-yl)vinyl)quinoline (1):** Compound **2** bromo(naphthalen-2-ylmethyl)triphenylphosphorane (2.25 g, 4.65 mmol) was dissolved in anhydrous tetrahydrofuran (10 mL) under Ar and the solution was cooled to 0 °C. Dry t-BuOK (1 g, 10.2 mmol) was added and stirred for 15 min. The solution of compound **3** 8-(hexyloxy)quinoline-2-carbaldehyde (1.29 g, 5 mmol) in dry THF (10 mL) was added dropwise into the reaction mixture. After addition, the mixture was stirred for 15 min. A few drops of water were added to quench the reaction. The mixture was extracted by CH_2_Cl_2_. The organic layer was washed with water for three times and dried over anhydrous Na_2_SO_4_. The solvent was removed in vacuo, the residue was purified by flash chromatography on silica gel using dichloromethane-petroleum ether (1:10) as the eluent to afford a white solid (1.5 g). Yield: 81%. Slow evaporation of compound **1** from dichloromethane/ethanol mixed solutions obtained light yellow block crystals of **1**. **^1^H NMR** (300 MHz, CDCl_3_, δ) 8.08 (d, *J* = 8 Hz, 1H), 7.97 (s, 1H), 7.85-7.80 (m, 5H), 7.73 (d, *J* = 8 Hz, 1H), 7.62-7.58 (m, 1H), 7.49-7.46 (m, 2H), 7.41-7.33(m, 2H), 7.06 (d, *J* = 8 Hz, 1H), 4.26 (t, *J* = 8 Hz, 2H), 2.10-2.07(m, 2H), 1.60-1.58 (m, 2H), 1.45-1.42(m,4H), 0.97-0.93(m, 3H). **^13^C NMR** (400 MHz, CDCl_3_, δ) 154.88, 140.44, 136.25, 134.33, 133.80, 133.65, 133.47, 130.11, 128.56, 128.45, 128.25, 127.92, 127.75, 126.40, 126.38, 126.29, 123.79, 119.47, 119.29, 109.41, 69.30, 31.75, 28.99, 25.81, 22.70, 14.12. **HRMS** (*m/z*): 382.2169 [M + H]^+^ (Calcd for C_27_H_27_NO: 382.2126).

**2. NMR, HRMS Spectra**


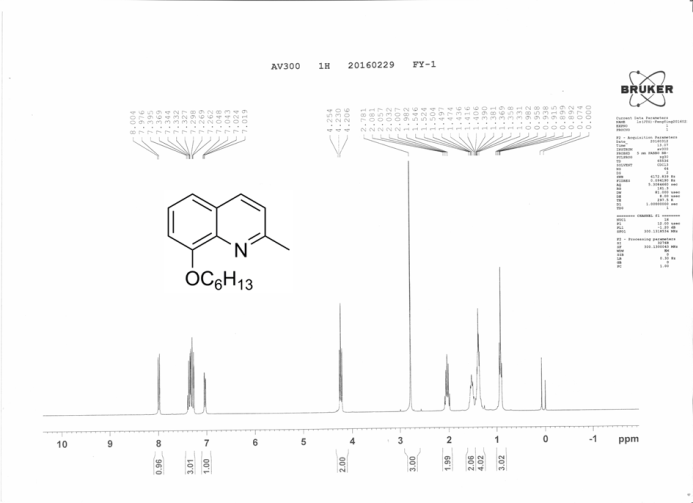


**Figure S1.** ^1^H NMR spectrum of 8-(hexyloxy)-2-methylquinoline


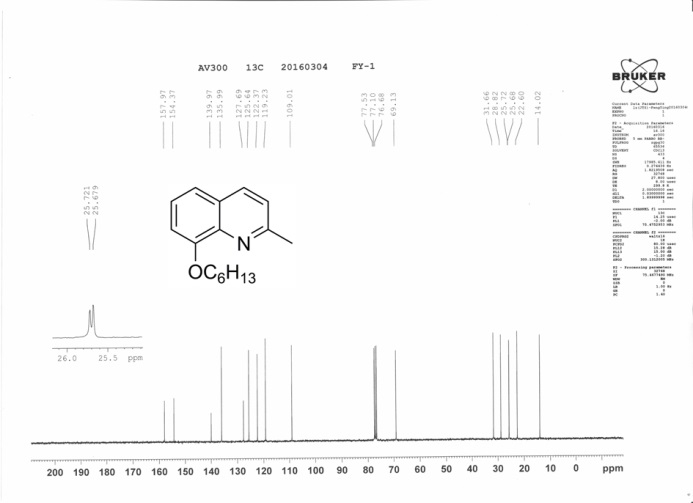


**Figure S2.** ^13^C NMR spectrum of (8-(hexyloxy)-2-methylquinoline


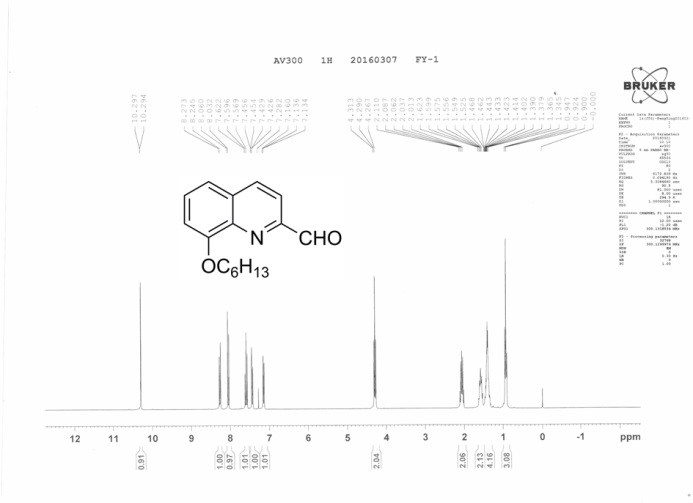


**Figure S3.** ^1^H NMR spectrum of 8-(hexyloxy)quinoline-2-carbaldehyde


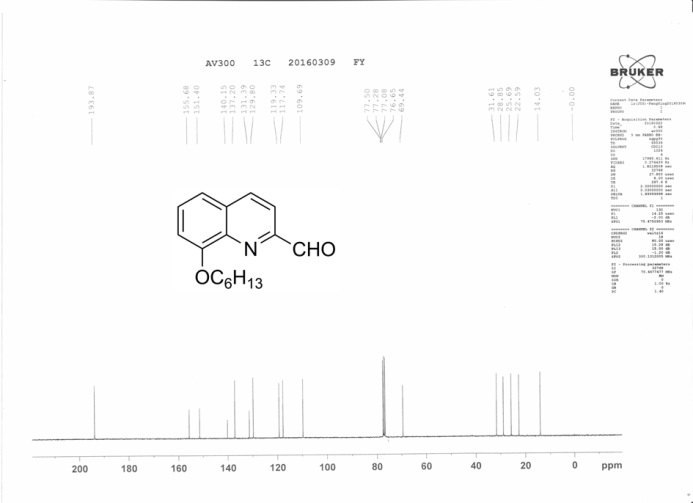


**Figure S4.** ^13^C NMR spectrum of 8-(hexyloxy)quinoline-2-carbaldehyde

**
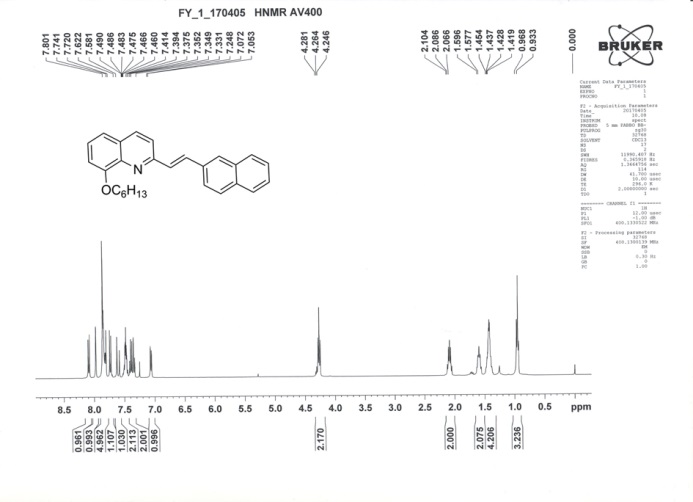
**

**Figure S5.** ^1^H NMR spectrum of (*Z*)-8-(hexyloxy)-2-(2-(naphthalen-2-yl)vinyl)quinoline

**
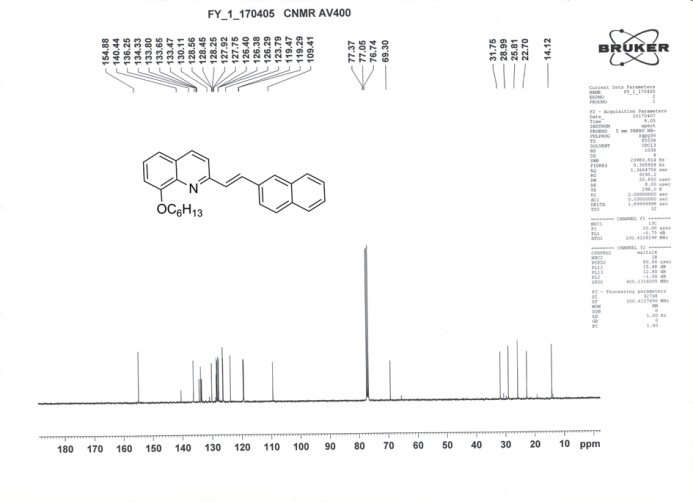
**

**Figure S6.** ^13^C NMR spectrum of (*Z*)-8-(hexyloxy)-2-(2-(naphthalen-2-yl)vinyl)quinoline

**
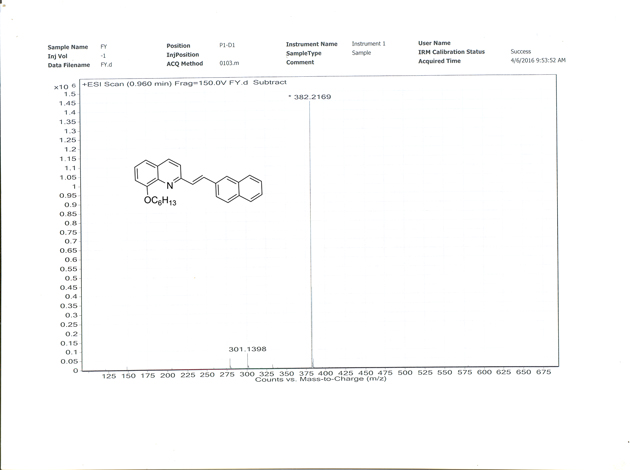
**

**Figure S7.** HRMS of compound (*Z*)-8-(hexyloxy)-2-(2-(naphthalen-2-yl)vinyl)quinoline
